# Assaying the Potency of Influenza Vaccines

**DOI:** 10.3390/vaccines3010090

**Published:** 2015-02-05

**Authors:** Philip D. Minor

**Affiliations:** National Institute for Biological Standards and Control/MHRA, Blanche Lane, Potters Bar, Hertfordshire EN6 3QG, UK; E-Mail: Philip.Minor@nibsc.org; Tel.: +44-1707-64-1000 (ext. 312); Fax: +44-1707-64-1366

**Keywords:** influenza vaccine, potency, SRD, reagent calibration, Influenza Collaborating Centres, Essential Regulatory Laboratories

## Abstract

The potency of vaccines must be determined to ensure that the appropriate dose is given. The manufacture and assessment of influenza vaccines are complicated by the continuously changing nature of the pathogen, which makes efficacy estimates difficult but also confounds attempts to produce a well-validated, consistent potency assay. Single radial diffusion has been used for decades and provides a relatively simple way to measure the amount of biologically active materials present in the vaccine. It requires reagents, which are updated on a regular, frequently yearly, basis and alternative methods continue to be sought.

## 1. Introduction

The potency of influenza vaccines has been successfully assayed by single radial immunodiffusion (SRID or SRD) for decades. Every year the circulating influenza viruses are reviewed and the strains included in the vaccines updated as necessary. This raises complex scientific and logistic issues for vaccine production, some of which are related to Single Radial Diffusion, the method for assessing potency. Attempts to develop alternative assays continue to be made.

Influenza viruses change continuously either by accumulating mutations in crucial sites, or through the exchange of entire segments of the genome. The key target for the protective immune response triggered by vaccination is directed is generally accepted to be the haemagglutinin (HA) although the neuraminidase (NA) may also contribute.

Classical vaccines against influenza are manufactured from virus grown in embryonated hens’ eggs. The non-replicating, non-adjuvanted vaccine that results may be whole virus, split virus or a purified subunit with all components other than the haemagglutinin and neuraminidase removed. Subunit vaccines are the major type in current use; although whole virus vaccines may generate a higher immune response, they are more reactogenic. The threat of H5N1 from 1997 [1,2] and the later reality of H1N1pdm in 2009 [3] renewed interest in the responses to vaccines [4] and the use of adjuvants [5,6,7,8,9,10]. Different vaccine types were investigated, including cell grown classical vaccines in addition to the one product already licensed [9,10,11], vaccines based on purified rDNA expressed protein [12] and others incorporating immune stimulatory molecules. All of these approaches have raised questions over the best way to assess potency. Moreover new methods for generating suitable strains by reverse genetics, including synthesis of the appropriate HA and NA genes for virus recovery, have potentially reduced the time taken to produce strains suitable for vaccine production, focusing attention on other parts of the process which are now rate limiting. This includes methods for assessing potency of the vaccines.

The dose of influenza vaccine given should be justified by assessing protection from disease in the human target species. In practice efficacy is very difficult to measure with any confidence [13,14]. Any general conclusion is made difficult by the continuously changing nature of the strains included in the vaccine or causing the epidemics, the age range of the vaccine recipients (who are mostly but not exclusively the elderly who respond differently to other age groups), and the administration of a booster dose every year, so that previous history of exposure to both vaccine and infection varies. Some doubt has even been expressed over the need to have a good antigenic match between the vaccine and the challenge virus [13], although this is contrary to the entire strategy that is currently followed. Assessments of the efficacy of influenza vaccines, where they have been done, have aggregated all classical vaccine products as if they were indistinguishable [13,14] which may not be the case [4,5,6,7,8,10,11]. On the other hand, it is not clear that the efficacy of vaccines from different manufacturers can be assessed and compared meaningfully through retrospective assessment of clinical records, because of the small numbers of patients and cases involved for any particular product. Vaccines from different sources clearly differ by* in vitro* tests (including single radial diffusion or SRD, the current potency test) but the significance of the differences, if any, is unknown. There are many unknowns in the area of influenza vaccines although there is a strong consensus that vaccination serves a useful function [13,14].

**Figure 1:**
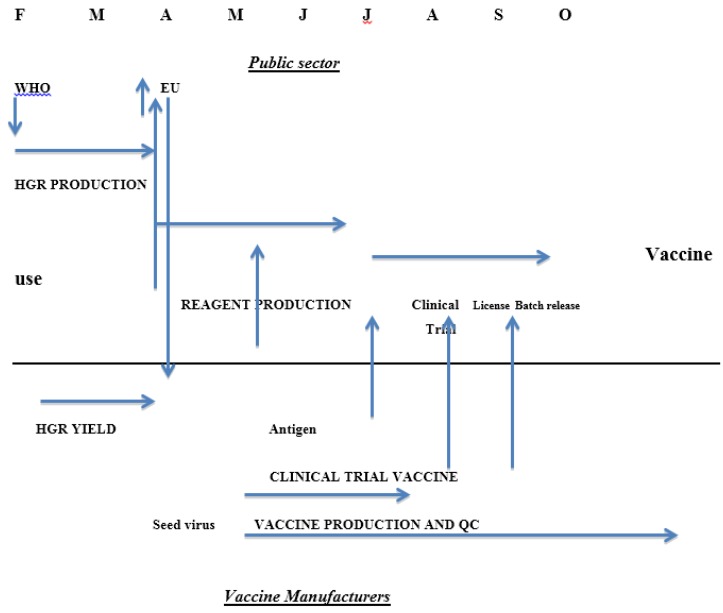
Calendar of influenza vaccine production in the European Union showing roles of the public sector bodies and vaccine manufacturers and the interactions between them.

Until recently there was a statutory requirement in the EU for assessment of serological responses by each manufacturer through a clinical immunogenicity study before a new season’s influenza vaccine was licensed. This requirement will be removed from 2015 [15] because it delays the process of getting vaccines onto the market (Figure 1) and the studies in small groups of healthy adults rather than the target groups had little or no benefit in assuring the quality of the vaccines. The studies were complicated in the same ways as studies of clinical efficacy: the age of the clinical trial participants was not the same as the common target group and the impact of previous exposure to vaccine or infection was not known. A further factor is the impossibility of developing reference materials to help in standardising serological assays every year for a continuously changing agent although the need is clear [16]. The difficulties encountered were mainly due to the changes in the strains of interest; influenza vaccine is essentially a new product every year in contrast to vaccines against other agents where the clinical properties of the product can be established with confidence over many years.

In principle, both the protective efficacy and immunogenicity of influenza vaccines could be assessed in suitable animal models if they were identified, but the method is slow and imprecise because of the relatively flat dose response curve and the variability inherent in animal studies. Measurement of influenza vaccine potency, therefore, involves the measurement of the amount of antigen in its native conformation on the assumption that, on average in a given population, this will correlate in a predictable manner with immunogenicity and protective efficacy. Antigenicity and immunogenicity are distinct properties and restricting the assay to the native form probably means they are linked for a particular strain and product. However, differences in the amount of antigen required to stimulate an immune response may occur between strains, some of which appear to be more immunogenic than others [4,5,6,7,8,10]. How much of the differences seen are really due to the virus as opposed to previous exposure of the study population to influenza is a matter for debate.

Antigen content was initially measured by agglutination assay [17]. The assay had a large number of uncontrollable variables, including the specific activity of the virus in agglutination of erythrocytes, which varies from strain to strain, and differences in the activity of whole virus, split and subunit preparations. The current regulatory requirement [18] is that there should be 15 micrograms of haemagglutinin protein in each dose of classical vaccine and the test used is SRD. It is likely that the immunogenic dose is age dependent; for example young children respond poorly to non-replicating influenza vaccines.

## 2. Measurement of Influenza Antigen Content by Single Radial Immunodiffusion

Single radial immunodiffusion (SRD or SRID) as a test for influenza vaccine antigen content was established and validated in the 1970s [17,19]. It replaced agglutination assays.

**Figure 2:**
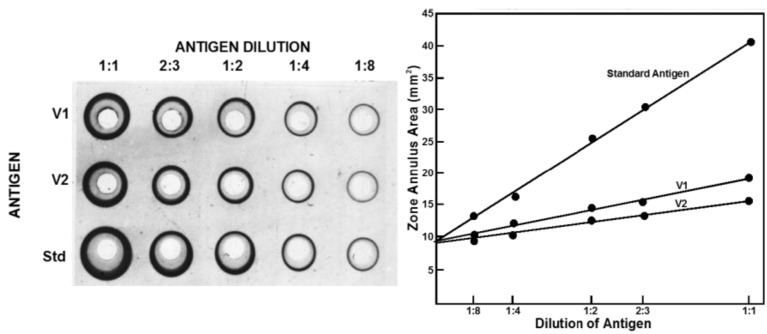
Single Radial Diffusion assay of influenza haemagglutinin. Stained plate showing zones and plot of zone size against dilution of analyte illustrating slope ratio statistical method for measuring potency.

SRD requires two reagents, an antibody reacting with the strain to be assayed and a reference antigen of known potency, which is homologous to the precise strain used in the vaccine. Wells are introduced into a slab of agarose containing the antibody at a concentration that will give zones of an appropriate size. Serial dilutions of the antigen to be assayed are then placed in the wells and the antigen diffuses until it reaches the zone of equivalence with the antibody dilution, where a precipitin ring forms. The agarose is washed free of excess antibody, dried and stained with Coomassie blue or some other appropriate agent to visualise the rings, whose size is measured. The greater the antigen content the larger the ring area. The reference is run in the same way and the ring sizes compared to measure antigen potency expressed relative to the reference. The potency of the reference is expressed in micrograms of haemagglutinin. The assay could involve choosing the dilutions used for the test and reference so that the ring sizes for both are in the same range and assessing the dose response statistically as a parallel line assay. Practically it is simpler to use the same dilutions for the test and reference and analyse the results by the ratio of the slopes of the dose response curves; the higher the initial content the steeper the slope as the test article is diluted. An example is shown in Figure 2.

While the assay is technically simple with no requirement for sophisticated equipment, its practical implementation is complex, because of the need for specific reagents and the variation in the strains included in the vaccine from year to year. In addition the WHO recommendations on strains to be included in the vaccine define viruses suitable for vaccine production as being “like” the prototype reference strains and manufacturers normally use modified laboratory strains, which grow to higher titres and are easier to process than the wild types; different modified strains may be used by different manufacturers. The antiserum is made by immunising sheep with haemagglutinin prepared from the WHO recommended strain or a derivative of it, whilst the antigen reference must be the strain used in the vaccine. Such is the scale of use of the reagents that the material used to produce the antigen reference can only be supplied by a manufacturer. The co-operative nature of reagent production and calibration and the need for rapid production if vaccine deadlines are to be met makes for a complex timetable.

## 3. Influenza Vaccine Timetable

The timetable for the EU in previous years is shown in Figure 1; clearly dropping the need for a clinical trial could shorten the process significantly [15].

The incidence of influenza in the world is reviewed formally twice a year at meetings convened by the World Health Organization (WHO), one in February for the Northern Hemisphere and the other in September for the Southern Hemisphere. Individual countries have National Influenza Centres who collate information from their country and send data and specimens to one of the five World Influenza Collaborating Centres recognised by WHO: CDC in the USA, NIMR in the UK, VIDRL in Australia, NIID in Japan and CDC in China. The data are reviewed to establish whether new strains are emerging and whether changes to the strains included in the vaccine are required. As the isolates tend to grow poorly in eggs, strains with better production characteristics are developed by re-assortment with a high yielding laboratory virus. These high growth reassortants consist of at least the HA and NA of the new circulating strain and the other viral proteins from the laboratory partner; they are examined to see if they are antigenically correct, and then sent to the manufacturers for assessment of their suitability for production. They are also sent to the Essential Regulatory Laboratories (ERLs) recognised by WHO: CBER in the USA, NIBSC in the United Kingdom, TGA in Australia and NIID in Japan. One major function of the ERLs is to develop and calibrate the reagents needed for measurement of vaccine potency. Typically reagents are available in early July, approximately four months after the decision taken in February. The process of preparing and calibrating the reagents is complex and requires close coordination between manufacturers and public sector bodies.

## 4. Preparation and Calibration of Reagents for SRD

The protocol for producing and calibrating reagents by the ERLs has been described by WHO [20]. One ERL takes the lead for a particular reagent set of antiserum and antigen. Sheep are immunised with haemagglutinin fragments produced by bromelain cleavage of purified virus to give a preparation lacking the transmembrane portion of the protein. The virus preparation used is either the wild type strain recommended by WHO or a derivative of it such as a high growth reassortant as it is generally believed that the immunogen used to produce the serum used in SRD does not need to be precisely the same strain as is used in the vaccine. Thus mixing wild type and reassortant HA as immunogens is acceptable, provided the resulting serum reacts well with all the vaccine haemagglutinins that it will be used to assay. This means that serum production can start before the manufacturers have identified their preferred strain. Consequently the production of the serum is usually not the rate-limiting step in reagent production it is claimed to be, although the process of immunisation can take eight weeks. Sheep are used because demand for the serum will be high and large volumes are needed. The immunization schedules are not fixed, being adapted empirically to give a high titre serum with good properties in the assay; some ERLs routinely pool serum from different animals, some increase the amount available by plasmaphoresis of the most suitable animal. There may be variations in the titre, cross reactivity and the quality of the SRD zones produced by the final product. However the serum does not need to be completely specific for native haemagglutinin as the concentration of serum can be adjusted to give a readable zone only with the native form.

In contrast to the serum the reference antigen must be made from the same strain as the vaccine whose potency is to be measured. If manufacturers use different reassortant strains to make vaccine, different antigen reagents will be required; moreover the amount used is such that the ERLs depend on the manufacturers’ production process to supply the raw material. Manufacturers must therefore establish their process with the new strain before production of antigen reagent can begin. This introduces delays.

The potency of the reagent and the vaccine will be expressed in micrograms so that there is some uniformity from year to year. The ERL taking the lead for a particular reagent makes or otherwise sources a whole virus preparation of the relevant influenza strain. This is termed the Primary Liquid Standard (PLS) and the protein concentration of the material and the proportion made up by the haemagglutinin are measured. Protein content can be determined by standard physico-chemical methods such as Lowry or commercial protein assays; some have used Kjeldahl nitrogen determination considering it more accurate and there is interest in using dilution Mass Spectrometry to give a more precise figure [21] The proportion of the total protein made up by haemagglutinin is determined by densitometric scanning of SDS PAGE gels. Modifications can include treating the protein with enzymes to degycosylate the haemagglutinin to improve the sharpness of the band and its separation from other components [22]. In the past the PLS was a live virus preparation, but it is increasingly inactivated by formalin or beta propiono lactone (BPL) treatment to make it easier to ship. The significance of this is that the native conformation of the haemagglutinin is easily disrupted, for instance by low pH which triggers a major structural rearrangement. It is likely that most or all of the HA in a live virus is in the native conformation; thus the microgram figure obtained probably represents total active haemagglutinin while inactivated preparations could theoretically include haemagglutinin denatured by the inactivation process. This would affect the calibration by overestimating the amount of active haemagglutinin present. The PLS is used to calibrate the antigen reference reagent in SRD. The antigen reference is a whole virus preparation made by a manufacturer and inactivated either with formalin or BPL and when the bulk is supplied the ERL will test it to evaluate the quality of the zones formed and roughly calibrate it against the PLS. The lead ERL will also determine an approximate range of concentrations for the serum in the agarose gel to give zones of suitable size and quality. The PLS and the candidate reagent are then shipped to the other ERLs who are all requested to measure the HA content of the PLS and use that to assign a figure for the HA content of the reagent; at least three ERLS including the lead need to provide data to allow an acceptable consensus figure to be reached. In addition manufacturers may be involved to provide an additional check.

The process serves to establish a consensus value that will be globally accepted. The antigen reagents involved in the calibration are all whole virus preparations of some sort whereas the vaccines that they are used to assay are nowadays almost all subunit preparations containing mostly the haemagglutinin and neuraminidase proteins. Detergent is included in the SRD assay that effectively makes the protein presentations similar in the different preparations.

## 5. Features of SRD

Firstly SRD assays for influenza vaccine antigens are mainly used for assessing the potency of the final product. The figure is expressed in micrograms so that there is comparability from year to year as the strains change, and batches from a single manufacturer or products from different manufacturer can be compared in a given year. As SRD has been used for decades there is a great deal of historic data and experience to fall back on which is valuable in an area as complex as influenza vaccine.

Secondly different treatments can result in a change in conformation and resulting loss of immunogenic activity of the haemagglutinin and strains can vary in their sensitivity to stress. An assay must therefore be able to assess stability; SRD measures antigen in the native conformation and is therefore suitable for this purpose. Assay after various treatments shows that there is a good correlation between antigen potency as measured by SRD and the induction of biologically active antibodies in mice, which does not exist for other types of assay that have been proposed, such as HPLC or other physico-chemical methods [23].

Thirdly, unlike established products where the process can be fixed in all significant respects and checked by a validated and constant assay, the changes in influenza strains mean in-process controls are required to assess yields and the quality of the product at various stages every year. Correctly calibrated reagents are unlikely to be available at the start of this process for all components. This leaves manufacturers in an insecure position and there is pressure to make the reagents available ever sooner. In practice an approximate figure has often been given before final calibration of the reagents to help early stage process development, or reagents from previous years can sometimes be used.

Given that it is intended to measure antigen content not protective efficacy (for reasons given earlier) the advantages of SRD include:

1. It measures the antigen thought to be most relevant to protection, specifically that in the native conformation.
2. It has been successfully used for decades so that there is a great deal of experience in its use and the issues that may arise as it is applied to an ever changing and complex product. Novel methods will almost certainly raise novel issues
3. It is of known acceptable accuracy and robustness as demonstrated by a number of unpublished proficiency testing studies by organisations such as EDQM (the European Directive for the Quality of Medicines which incorporates the European Pharmacopoeia) Agreement between competent laboratories assaying the same material is of the order of 5%
4. It is by its nature specific for the type and subtype of virus to be assayed, for example H1N1 or H3N2 or B. There may be issues where more than one strain of a particular subtype is included as where B strains of the Yamagata and Victoria lineage are both present and there is extensive cross reaction between them but it can be used to assay the potency of the final finished product which is almost always multivalent.
5. It is simple in principle and in application so that sophisticated equipment is not needed and the assay is both flexible and practical. It could be applied to developing country manufacture.

The disadvantages of SRD include:

1. The production of calibrated reagents takes time; typically the strain selection process in the Northern Hemisphere takes place in February and the final calibrated reagents are released by late June to early July (Table 1) approximately four months later. Process validation by the manufacturer therefore cannot usually be done with fully calibrated reagents. This has been put forward as a major obstacle to the timely production of vaccines in response to the 2009 H1N1 pandemic. Analysis of the events and time course of the global response suggest that while the reagents were not immediately available, other factors were at least equally significant, including the timing of the difficult decision to declare the pandemic so that manufacturers could commit to changing production to the new pandemic strains.
2. The reagents and therefore the assays vary from year to year and are not in the full control of either the manufacturers (who depend on the ERLs for calibrated reagents) or the ERLs (who depend on the manufacturers to supply the antigen that will be used to prepare the reagents). This leaves both in vulnerable positions.
3. Technically, the dynamic range of the assay is limited, covering 7.5 to 30 micrograms per dose. Where low dose preparations are proposed in pandemics to spare antigen, this has posed a problem. Similarly adjuvants can interfere with the assay although this is not necessarily the case; for example SRD fails to work with alumn-based adjuvants although it will give reliable figures with some squalene-based adjuvants.
4. There is a philosophical issue relating to the metrological credibility of the process. All masses expressed in kilograms or its subdivisions are traceable to the standard held in Paris at BIPM, and the process is very sophisticated if done rigorously, including confidence intervals on the accuracy or uncertainty of the measurement. Micrograms of influenza antigen are not traceable in this way and refer only to the mass present in a particular conformation. While this may seem sophistry, it means that the accuracy of the calibration is likely to change from year to year. In particular if a new reagent is required because old stocks are exhausted it will not be identical to the old stock. Usually such differences are expected to fall within the experimental error of the assay or the range of the specifications set; however it is advisable to use one set of reagents throughout one season to avoid discontinuities in the assay results.
5. The assay is designed and has been used to assay vaccines derived from virus grown in embryonated hens’ eggs or in cell culture [12]. Novel vaccines have been developed, including antigens expressed in plants or insect cells or which include immunostimulatory molecules where SRD is not satisfactory. SRD has never been appropriate for live attenuated influenza vaccines where assays of infectivity are applied.

Despite the years of use of SRD unexpected findings still arise. It is a common experience that some materials give well-defined tight zones whereas others that are nominally the same give hazy or mixed zones. This is assumed to be attributable to process differences. However it is not a consistent feature for a particular producer, being seen with some strains but not others. It is also assumed that the serum used does not affect the quantitative result obtained provided satisfactory zones are produced and the antigen assayed is homologous to the reference. However there have been instances where a change in the serum resulted in a real and consistent change in the potencies recorded by about 10%. This suggests that the reference antigen and the tested vaccine were not identical despite being produced from the same strain and hgr presumably as in the other example mentioned above because of process differences. Subtle differences in products and strains are detectable by SRD that can affect the potencies recorded. This is unlikely to be just a feature of SRD and should be borne in mind when evaluating alternative methods where there is currently far less experience.

**Table 1:** Dates of selection of new vaccine strains by WHO and the availability of calibrated reagents.

Date of Selection	Type	Production Strain	Date of Release of Calibrated Reagent
24 Feb 2006	H3N2	IVR-142	23 June 2006
		NYMX 161B	06 July 2006
16 Feb 2007	H1N1	IVR-145	30 June 2007
20 Feb 2008	H1N1	IVR-148	8 June 2008
	H3N2	NYMCK 175	13 June 2008
16 Feb 2009	B	B Bris/60	11 June 2009
1 March 2010	H3N2	NYMCX 183	26 May 2010
		NYMCX 187	26 May 2010
	B	NYMC BX35	28 May 2010
5 April 2011	No changes		
8 Feb 2012	B	NYMC BX39	20 June 2012
	H3N2	IVR-165	20 June 2012

## 6. Alternative Assays to SRD

The properties of an ideal assay for influenza vaccine would include the following.

1. It would measure a biologically relevant parameter to give meaningful measurements of potency, stability and clinical effect.
2. It would be accurate and precise although the degree of accuracy and precision required is not specified.
3. It would be rapid to allow real time monitoring of processes.
4. It would be specific at least to the level of virus type and subtype.
5. It should have a wide dynamic range so as to measure low dosage forms and it should work on different vaccine types such as might be used in pandemics.
6. It would either need
  (a) no specific reagents
  (b) reagents that do not require changing every time there was a strain change or
  (c) reagents that are specific, but easier to make, for instance if they could be made in small quantities not requiring production at manufacturing scale.
7. It should be readily applicable in developing countries.

Single Radial Diffusion clearly meets points 1, 4, and 7, and arguably 2 and 3 depending on the precision and speed needed. Its dynamic range and the need for reagents are issues although they have been successfully dealt with for decades.

A number of alternative assays have been suggested. None are approved for use by regulatory agencies as yet and the following is almost certainly not a comprehensive list of assays under investigation. The intent is briefly to indicate the types of approach being taken and their strengths and weaknesses.

1. HPLC [24]. HPLC is a well-established technique for separating and quantitating proteins. In the form applied it measures denatured protein rather than the biologically active form but for a well-established process the total protein and the amount of the active form are likely to be fairly closely related. Thus, in the early stages of process evaluation, where yields are being examined, the total protein is likely to be a very useful parameter. HPLC is unlikely to be fully acceptable at the point of release or in stability studies because it does not measure protein in a biologically relevant conformation. It can be accurate and precise, and rapid enough to allow real time monitoring of processes although the dynamic range is not great. There may be an issue in separating the haemagglutinins from different vaccine strains so that applying it to trivalent or tetravalent bulks will not be straightforward. HPLC is a widely used technique throughout the world. Finally accurate quantitation and identification is likely to require a homologous calibrated reference material. HPLC is therefore a useful adjunct to SRD but not a replacement; it is not capable of demonstrating stability except where the instability is due to protein degradation. It is possible that a preparation could be treated to remove the denatured form of the HA protein and the remainder quantitated by HPLC, which would make it stability indicating
2. Mass spectroscopy [21]. By including a formally calibrated isotopically labelled peptide of a sequence derived from the haemagglutinin and enzymatically digesting a mixture of the peptide and the haemagglutinin the relative size of the peptide and HA derived peaks can give a very accurate figure for the amount of HA present. In this form it does not measure conformationally correct protein content but could be used to assay the protein content of the primary liquid standard (PLS) used in calibrating reagents. Currently it is unlikely to be an acceptable test for the final product but it could be a useful addition to the methods used by the ERLs in calibrating the reagents.
3. A modification of Mass spectroscopy involves capturing the active form of the HA by specific antibodies linked to magnetic beads and measuring the amount caught by mass spectroscopy. As specific antibodies are used the method can be used on formulated final vaccine and measure a biologically relevant protein. The results would be expressed in absolute terms without the need for a homologous influenza virus reference although it does require specific antibodies. The equipment is also potentially not yet wide spread.
4. ELISA. Various ELISA formats have been proposed:
  (a) The first [25] involves antibodies recognising non-conformational epitopes that will measure total protein but not the biologically active form.
  (b) An approach to measuring the biologically active form is to develop a panel of monoclonal antibodies with neutralising or haemagglutination inhibiting activity for a particular strain. The assay therefore measures a biologically relevant property. As described by Bodle *et al*. [26] it is also very accurate, precise and rapid with a far wider dynamic range than SRD. It is specific by its very nature, can in principle be applied to a multivalent product and the general technique is very widely used across the globe and therefore readily applicable in many settings. In addition when a new strain emerges by mutation, as is the usual seasonal pattern, it will not have mutated all epitopes. Some members of the antibody panel should still react and can be used to measure the amount of haemagglutinin in the new strain. There is therefore no need to generate a new serum immediately. It also seems plausible that the new virus could be measured by reference to an old antigen reagent if the monoclonal antibody reacts equally well with both, so no new reagents would be needed for that season at all although the panel would have to be kept up to date and monitored closely. However Bodle *et al.* who described the approach [26] report differences in avidity of monoclonal antibodies for haemagglutinins from different strains with which they nonetheless react. This is very likely to affect the potencies measured, so that it is probable that a homologous reference reagent will still be required. It is also not clear how much would be required.
  (c) A version of this approach is being developed by InDvr [27] in which panels of antibodies are included on a chip, which can be used to assay all haemagglutinins present in the vaccine at once. Another version of the same approach has also been described [28]. This requires further evaluation from the point of view of the amount of reference antigen required and its suitability for use over multiple seasons. If only a small amount of antigen is required a batch could be made in house by the ERLs so that development of the process at the manufacturing site will not be required and time will be saved. The reagent would still require calibration by a collaborative study among the ERLs however.
  (d) A related alternative to the use of a panel of antibodies directed at the variable globular head of the HA is the use of conformation dependent cross-reactive antibodies directed at the stalk region [29,30]. These are being investigated in the same way [31]. An ELISA based on attachment of the native conformation to synthetic receptors has been described which would not require specific reagents provided the avidity was constant between strains [32]
5. Antibody dependent Surface Plasmon Resonance [33]. When an incident beam of p-polarized light strikes an electrically conducting gold layer at the interface of a glass sensor with high RI (Refractive Index) and a medium with low RI at a given angle, excitation of surface plasmons takes place and the intensity of the reflected light is reduced. A slight change at the interface, for instance binding of an antibody or binding of an antigen to an immobilised antibody leads to a change in SPR signal. Binding can therefore be measured precisely on very small quantities of material in a strain or type specific manner. The method would require both antibodies and reference antigen, but use of reagents may be small so that small batches produced at the ERLs are a possibility. The same considerations apply as to the ELISA approaches outlined above.
6. A modification of one of the ELISA methods uses SPR to detect binding rather than ELISA by coating the chip with synthetic glycans linked to sialic acid imitating the influenza receptor. This has the advantage of eliminating the need for an antibody reagent and using the same glycan reagent year on year. It requires a reference antigen but possibly not in large amounts as above. It has been successfully applied to a number of strains [34] It is rapid, accurate, relates to SRD and assays only the active form of the HA. The fact that it uses binding to the receptor that all influenza viruses use means that it cannot be used to assay multivalent products as all will bind. It is also possible that differences in the avidity of binding could affect the use of the method. The equipment is currently sophisticated and expensive.

The above list is not intended to be comprehensive. There are clearly a number of assay methods under investigation that may be applicable for some immediate usages (such as in process validation by HPLC where a great deal of information can be gained and validated with other methods later if needed) and others where it appears that there may be advantages in terms of reagent needs compared to SRD. Over the past decades there have been several attempts to develop replacements for SRD, which have not been successful. Experience has revealed many unexpected pitfalls in the assay of influenza haemagglutinin and any new method is also likely to have currently unrecognised issues. For instance the use of a common set of references over a period of several years would be a major advantage in terms of early availability, and the investigation of ELISA based assays is producing interesting results. However, the changes in avidity despite unchanged epitopes sounds a cautionary note and clearly much further developmental work is required. This may have an impact on manufacturer and regulator willingness to change the assay, which is likely to involve a great deal of validation and expense. A number of developments in the system have emerged; the use of reverse genetics and synthetic genes may accelerate the preparation of production strains where needed. It has been proposed that protein expressed in bacterial systems is satisfactory for preparing antiserum reagents by inducing antibodies in sheep particularly where there are difficulties in preparing the material from virus [35].

## 7. Conclusions

The potency of influenza vaccine is currently expressed in terms of the amount of haemagglutinin in the correct antigenic conformation. This is believed to correlate with immunogenicity at least for a given strain and is currently determined by single radial immunodiffusion (SRD or SRID) a technique that is simple in principle but which demands the production of calibrated reference materials on an annual basis.

The system for manufacture of influenza vaccines as currently constituted is complex and cooperative and SRD is part of that complexity. This is perhaps a good reason to alter it to something more straightforward but is also very good reason to do so with great caution.
